# FGFR inhibitors combined with nab-paclitaxel - A promising strategy to treat non-small cell lung cancer and overcome resistance

**DOI:** 10.3389/fonc.2023.1088444

**Published:** 2023-02-10

**Authors:** Feng Ma, Xinhai Zhu, Yuchun Niu, Aitao Nai, Shoaib Bashir, Yan Xiong, Yunlong Dong, Yin Li, Jian Song, Meng Xu

**Affiliations:** ^1^ Department of Oncology, The First Affiliated Hospital of Jinan University, Jinan University, Guangzhou, China; ^2^ Department of Oncology, The First Affiliated Hospital of Hebei North University, Zhangjiakou, China; ^3^ Department of Radiation Oncology, The First People’s Hospital of Foshan, Foshan, China; ^4^ Department of Oncology, The First Affiliated Hospital of Nanhua University, Hengyang, China; ^5^ Guangzhou Institute of Respiratory Health, The First Affiliated Hospital of Guangzhou Medical University, Guangzhou, China; ^6^ Department of Thoracic Surgery, Tianjin Baodi Hospital, Baodi Clinical College of Tianjin Medical University, Tianjin, China; ^7^ Department of Oncology, Zhongshan Torch Development Zone People’s Hospital, Zhongshan, China

**Keywords:** FGFR inhibitor, AZD4547, nab-paclitaxel, MAPK signaling pathway, NSCLC, drug sensitivity

## Abstract

Lung cancer has high morbidity and mortality rates worldwide, and NSCLC accounts for 85% of all lung cancer cases. Despite the development of targeted therapies and immunotherapy, many NSCLC patients do not effectively respond to treatment, and new treatment strategies are urgently needed. Aberrant activation of the FGFR signaling pathway is closely related to the initiation and progression of tumors. AZD4547, which is a selective inhibitor of FGFR 1-3, can suppress the growth of tumor cells with deregulated FGFR expression *in vivo* and *in vitro*. However, further exploration is needed to determine whether AZD4547 can play an antiproliferative role in tumor cells without deregulated FGFR expression. We investigated the antiproliferative effect of AZD4547 on NSCLC cells without deregulated FGFR expression. *In vivo* and *in vitro* experiments showed that AZD4547 exerted a weak antiproliferative effect on NSCLC cells without deregulated FGFR expression, but it significantly enhanced the sensitivity of NSCLC cells to nab-paclitaxel. We found that AZD4547 combined with nab-paclitaxel suppressed the phosphorylation of the MAPK signaling pathway, led to cell cycle arrest in the G2/M phase, promoted apoptosis, and inhibited cell proliferation more substantially than nab-paclitaxel alone. These findings provide insight into the rational use of FGFR inhibitors and personalized treatment of NSCLC patients.

## Introduction

Non-small cell lung cancer (NSCLC), which is a major subtype of lung cancer, is one of the most commonly diagnosed cancers worldwide, and 2.2 million new NSCLC cases and 1.8 million NSCLC-related deaths were recorded in 2020. Although the incidence rate of NSCLC has decreased and is now ranked second, the mortality rate of NSCLC is still the highest among cancers (1). The development of targeted therapies using tyrosine kinase inhibitors and immunotherapies has significantly improved the quality of life of NSCLC patients. However, NSCLC patients still face many challenges. Regardless of the treatment that is administered, most patients eventually experience disease progression, and additional treatment options are required for these patients.

Fibroblast growth factors (FGFs) are a family of proteins that play crucial roles in a variety of biological processes, including embryonic development, metabolism, tissue homeostasis, cancer, wound repair, and angiogenesis. FGFs modulate a wide range of cellular processes, including cell proliferation, differentiation, survival, migration, and metabolism, by binding to and activating their receptors. In mammals, the FGF receptor (FGFR) family comprises 5 prominent members, FGFR1-4 and FGFRL1, which is a truncated FGFR that lacks an intracellular domain. Depending on the contents of different cells and tissues, the typical signaling pathways that are downstream of FGF/FGFR interactions include the Ras/Raf-MEK-MAPK (mitogen-activated protein kinase), phosphatidylinositol-3 kinase/protein kinase B (PI3K/AKT), PLCγ, and signal transducer and activator of transcription (STAT) pathways (2).

AZD4547, which is a selective inhibitor of FGFR 1-3, inhibits FGFR signaling and the growth of tumor cell lines with deregulated FGFR expression. Preclinical studies have demonstrated that AZD4547 has potent activity against tumors with deregulated FGFR expression. However, AZD4547 has no effect against over 100 additional tumor cell lines, showing that the *in vitro* antiproliferative effects of AZD4547 is limited to tumor cell lines with deregulated FGFR expression (3). This also means that most clinical patients may not respond well to FGFR inhibitors. Recent clinical trials have also confirmed this result: two phase I/II studies of pancancer patients showed that patients with FGFR fusions/rearrangements had the highest response rate, followed by those with FGFR mutations, whereas patients with other alterations or no FGF/FGFR alterations rarely had responses or had no response (4, 5). Additionally, many studies have shown that AZD4547 does not exhibit efficacy in patients with FGFR amplification, including in lung cancer patients (6–9). Therefore, it is necessary to further explore the antiproliferative effect of AZD4547 on tumor cells, especially on tumor cells without deregulated FGFR expression, so that it can be used more rationally.

Nanoparticle albumin-bound paclitaxel (nab-PTX) was approved by the United States Food and Drug Administration for the treatment of metastatic NSCLC in 2005. Nab-PTX plus carboplatin can be considered as a first-line treatment for patients with advanced NSCLC. Studies have confirmed that nab-PTX is more effective than conventional solvent-based paclitaxel (10). However, drug resistance is inevitable, regardless of whether nab-PTX is used for targeted therapy or chemotherapy.

Combination treatment is considered to be a reasonable and feasible approach to achieve optimal therapeutic effects. Combination therapies that target multiple pathways have become preferred choices for cancer treatment, and such treatments can effectively improve treatment efficacy, overcome drug resistance, and expand the application range of drugs. For example, bevacizumab, a humanized monoclonal antibody with a high binding affinity for circulating vascular endothelial growth factor A, has been demonstrated to enhance the response rate, progression-free survival, and survival of patients with advanced nonsquamous NSCLC when added to various chemotherapeutic regimens (11). The combination of pembrolizumab and chemotherapy enables patients with a PD-L1 tumor proportion score lower than 1%, a population with little chance of benefitting from single-drug PD-1 and PD-L1 inhibitors, to obtain survival benefits (12). Similarly, although the single-drug data for AZD4547 in ER-positive breast cancer have shown very low efficacy, in patients with ER-positive breast cancer whose disease has progressed on aromatase inhibitors, the addition of this FGFR inhibitor yields a clinical benefit, irrespective of FGFR amplification (13). Therefore, a combination strategy involving FGFR inhibitors may be appropriate for the treatment of NSCLC.

Our research shows that AZD4547 exerted a weak antiproliferative effect on NSCLC cells without deregulated FGFR expression. However, the combination of AZD4547 and nab-PTX suppressed the phosphorylation of the MAPK signaling pathway, which led to cell cycle arrest in the G2/M phase, promoted apoptosis, and exerted a synergistic antitumor effect. Our findings provide a promising new strategy to treat non-small cell lung cancer and overcome resistance.

## Materials and methods

### Cell culture, reagents, and antibodies

The A549 and PC9 human NSCLC cell lines were purchased from the Cell Bank of the Type Culture Collection of the Chinese Academy of Sciences (Shanghai, China). The cells were cultured in Roswell Park Memorial Institute 1640 medium supplemented with 10% fetal bovine serum (FBS, Gibco) and 1% penicillin-streptomycin at 37°C in an atmosphere of 5% CO2 and 95% air. The cell lines used in this study were not contaminated by mycoplasma. AZD4547 was purchased from SelleckChem, USA. Nab-PTX was purchased from CSPC Pharmaceutical Group Limited. The GAPDH (10494-1-AP) antibody was obtained from Proteintech Group. The FGF2 (DF6038), ERK1/2 (AF0155), phospho-ERK1/2 (AF1015), JNK 1/2/3 (AF6318), phospho-JNK 1/2/3 (AF3318), p38 (AF6456), and phospho-p38 (AF4001) antibodies were obtained from Affinity Biosciences. The Cleaved Caspase3 (9664), Bax (41162), Bcl-2 (4223), EREG (12048), HSP70 (4873), phospho-Jun (3270), and phospho-p53 (82530) antibodies were obtained from Cell Signaling Technology.

### Cell viability and colony formation assays

The cell viability assay was performed using Cell Counting Kit-8 (catalog KGA317; KeyGEN, Nanjing, China) according to the manufacturer’s instructions. Cells (5 × 10^3^ cells/well) were seeded in 96-well plates and cultured overnight. The next day, the cells were treated with different concentrations of drugs for different times. The IC50 was calculated, and cell viability curves were plotted using GraphPad Prism 8.0 (GraphPad Software, La Jolla, CA, USA).

For colony formation assays, a single-cell suspension was seeded in six-well plates at a density of 300 cells per well. Two weeks later, the colonies in each well were fixed in 4% paraformaldehyde (Solarbio, P1110) and stained with 0.5% crystal violet (Sigma Aldrich, St. Louis, MO, USA). The number of stained colonies was counted. All analyses were carried out in triplicate.

### Flow cytometric analysis

For the apoptosis and cell cycle assays, cells were incubated with or without drugs for 24 h before being harvested. Cell apoptosis assays were conducted by using an Annexin V-FITC/PI apoptosis detection kit (catalog KGA107; KeyGEN, Nanjing, China) according to the manufacturer’s protocol. For the cell cycle assay, cells were harvested and fixed with 70% ethanol at 4°C for 16 h and then stained with propidium iodide (catalog R37108; Thermo Fisher Scientific) in the presence of RNase A. Final acquisition and analysis were performed using a FACScan (BD Biosciences).

### RNA extraction and qPCR assays

Total RNA was extracted from cultured cells using TRIzol reagent (Invitrogen, Shanghai, China) according to the manufacturer’s instructions, and then, the total RNA concentration was determined using a Nanodrop 2000 (Thermo). Total RNA (1,000 ng per sample) was reverse transcribed in a final volume of 20 μl using random primers under standard conditions for the FastKing RT Kit (catalog KR116; Tiangen Biotech, Beijing, China). PCR analysis was performed using SYBR Green Talent qPCR PreMix (catalog FP209; Tiangen Biotech, Beijing, China), and the levels of the amplified PCR products were quantified and normalized using the housekeeping gene GAPDH as a control. Quantitative PCR (qPCR) and data collection were carried out using a Bio-Rad CFX connect real-time PCR system (Bio-Rad Laboratories, USA). The primers for the genes of interest were synthesized by Sangon Biotech (Shanghai, China). The sequences for the gene-specific primers used in this study were as follows: FGF2-F: GACCCTCACATCAAGCTACAA, FGF2-R: AGCCAGTAATCTTCCATCT TCC, EREG-F: GCTCTGACATGAATGGCTATTG, EREG-R: AGTATAACCCACTT CACACCTG, HSP70-F: CCTACTCTTGTGTGGGTGTTT, HSP70-R: ACCGTTCA GTGTCCGTAAAG, GAPDH-F: AAATCAAGTGGGGCGATGCT, and GAPDH-R: CAA A TGAGCCCCAGCCTTCT.

### Western blotting

Briefly, the indicated cells were lysed using RIPA buffer (Beyotime, China) supplemented with a protease and phosphatase inhibitor cocktail (CWBIO, Beijing, China). The concentration of the isolated protein was quantified using a BCA kit (CWBIO, Beijing, China). The proteins were separated by SDS−PAGE and transferred to nitrocellulose membranes (Millipore Sigma, USA). The membranes were blocked and then incubated overnight at 4°C with the indicated primary antibodies in primary antibody dilution buffer (Beyotime, China). After washing three times, the membranes were incubated with the indicated secondary antibodies for 1 h at room temperature. Immune complexes were detected using an enhanced chemiluminescence (ECL) kit (Millipore Sigma, USA).

### Tumor xenograft assay

Research involving animals was performed in strict accordance with the policies of the animal ethics committee of the Southern Medical University of China. The female BALB/c nude mice (BALB/c 4–5 weeks old) that were used for the experiments were randomly divided into the indicated groups (6–8 mice/group) before inoculation. As indicated, 5 × 10^6^ cancer cells in 0.15 ml PBS per site were subcutaneously injected into the right flank of each mouse. The tumors were measured every week, and the tumor volumes were calculated using the following formula: ((major axis) × (minor axis)2)/2. PBS, AZD4547, nab-PTX, and AZD4547 plus nab-PTX were injected into the mice in the four groups once per week for 3 weeks. The mice were sacrificed at the endpoint, and the tumor volume and weight were measured individually.

### RNA-sequencing

Total RNA was extracted from A549 cells of different treatment groups using TRIzol and quantified using a NanoDrop (Thermo). RNA samples (1 μg) were treated with VAHTS mRNA capture beads (Vazyme, China) to enrich poly A+ RNA. The VAHTS Universal V8 RNA-seq Library Prep Kit for Illumina (Vazyme, China) was used to prepare the RNA-seq libraries according to the manufacturer’s instructions. Briefly, approximately 100 ng of poly A+ RNA samples were fragmented and reverse transcribed into double-stranded cDNA. Then, these cDNA fragments were subjected to end repair, adenine tail addition, and adaptor ligation. The purified products were subjected to 12 cycles of PCR amplification to generate the final cDNA libraries. All library sizes and concentrations were confirmed on the Agilent 4200 TapeStation System and a Qubit 3.0 Fluorometer (Thermo). Finally, the libraries were sequenced on an Illumina NovaSeq 6000 by Haplox Biotechnology Co (Shenzhen, China).

### Bioinformatics analysis

Analysis was performed using Metascape (http://metascape.org), which is an integrated and user-friendly web tool that was designed to facilitate multiplatform OMICs data analysis and interpretation for the experimental research community. The generic analysis workflow consists of four major components: identifier conversion, gene annotation, membership search, and enrichment analysis (14).

### Statistical analysis

The results were analyzed using GraphPad Prism 8.0, and the data are presented as the means ± standard deviations (SDs). Statistical analyses were performed using Student’s t test or one-way ANOVA with Tukey’s multiple comparisons test if there were more than two groups. P<0.05 was considered to indicate statistical significance. Throughout this study, *: p< 0.05; **: p< 0.01; ***: p< 0.001, ****: p< 0.0001.

## Results

### AZD4547 combined with nab-PTX significantly inhibited cell proliferation *in vivo* and *in vitro*

**Figure 1:**
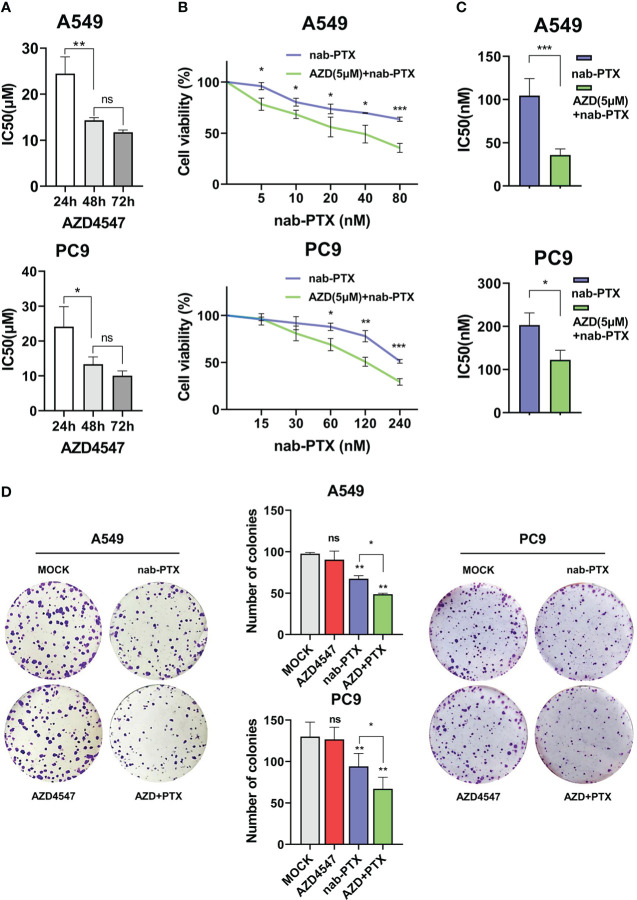
AZD4547 combined with nab-PTX significantly inhibited cell proliferation *in vitro*. **(A)** IC50 of AZD4547 in A549 and PC9 cells at different time points. **(B, C)** Cells cotreated with 5 μM AZD4547 and varying concentrations of nab-PTX showed larger reductions in proliferation than cells treated with nab-PTX alone. **(D)** Colony formation assay. Cells cotreated with AZD4547 (5 μM) and nab-PTX (A549 cells 20 nM, PC9 cells 60 nM) exhibited significantly less colony formation than cells treated with nab-PTX alone. No significant change was observed in cells treated with AZD4547 alone. ns: no significance, *: p< 0.05, **: p< 0.01, ***: p< 0.001.

**Figure 2:**
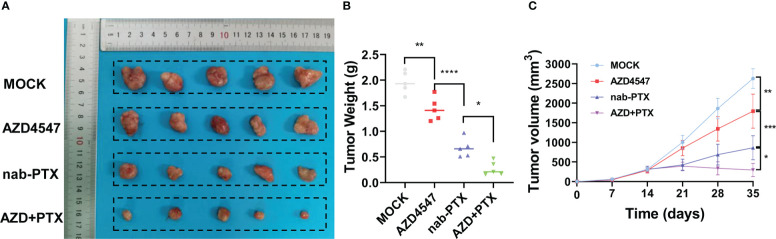
AZD4547 combined with nab-PTX exerted a synergistic antitumor effect *in vivo*. **(A)** Representative images of xenograft tumors formed by PC9 cells in nude mice. **(B)** The tumors were excised and weighed. **(C)** Cells were subcutaneously injected into nude mice, and tumor growth was assessed every week. *: p< 0.05, **: p< 0.01, ***: p< 0.001, ****: p< 0.0001.

First, we measured the effects of AZD4547 on the survival of A549 and PC9 cells when administered at different concentrations and for different times (**Figure 1A**). We found that 5 μM AZD4547 had no significant effect on cell viability, but when it was combined with nab-PTX, the cell viability of A549 and PC9 cells decreased significantly (**Figure 1B**). Next, A549 and PC9 cells were cotreated with 5 μM AZD4547 and nab-PTX for 24 h. Cotreatment of cells with AZD4547 (5 μM) and nab-PTX resulted in nab-PTX IC50 values of 35.78 nM (A549 cells) and 122.5 nM (PC9 cells), indicating higher sensitivity in these cells than in those treated with nab-PTX alone (IC50 = 144.4 nM (A549 cells) and 202.9 nM (PC9 cells)) (**Figure 1C**). In addition, colony formation assays revealed that the inhibitory effect of AZD4547 combined with nab-PTX on colony formation was significantly stronger than that of nab-PTX alone (**Figure 1D**). To further explore the inhibitory effect of AZD4547 combined with nab-PTX on tumor growth *in vivo*, we conducted a tumor xenotransplantation experiment in nude mice (**Figure 2A**). The result showed that tumor growth was strongly inhibited in the combined treatment group compared with the other groups (**Figures 2B, C**). Our results indicate that the combination of AZD4547 with nab-PTX has a better effect than treatment with nab-PTX alone and exerts synergistic antitumor effects.

### The effect of AZD4547 combined with nab-PTX on cell cycle progression and apoptosis

**Figure 3:**
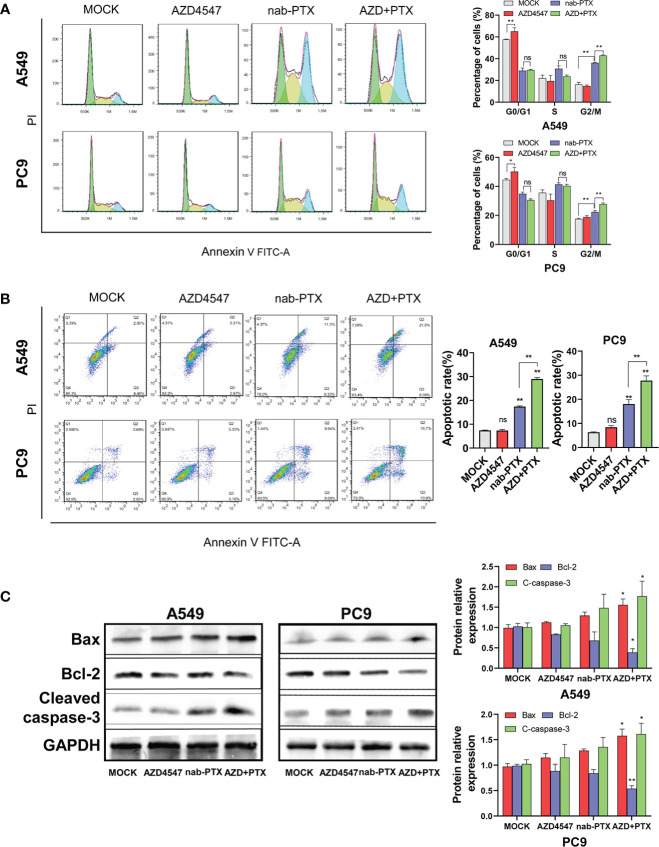
Cell cycle and apoptosis analysis. **(A)** Cells cotreated with AZD4547 and nab-PTX exhibited a significantly higher rate of apoptosis than those treated with nab-PTX alone. (Left: Representative FACS profiles are shown, and the cell population in the Annexin V-FITC/PI quadrant represents apoptotic cells. Right: The percentage of apoptotic cells is shown in histogram form). **(B)** Cell cycle arrest in the G2/M phase was more evident when AZD4547 was combined with nab-PTX than when nab-PTX was used alone (Left: Cell cycle distribution of cells after different treatments for 24 h. Right: Histogram showing the percentage of cells in each phase of the cell cycle). **(C)** AZD4547 regulated the expression of the apoptosis-associated proteins Bax, Bcl-2, and cleaved caspase-3. ns, no significance, *: p< 0.05, **: p< 0.01.

We next used flow cytometry to detect the effect of AZD4547 combined with nab-PTX on cell cycle progression and apoptosis by flow cytometry. In the cell cycle analysis, AZD4547 and nab-PTX blocked the cell cycle in the G1 and G2/M phases, respectively. When AZD4547 and nab-PTX were combined, significantly more cells exhibited cell cycle arrest in the G2/M phase (**Figure 3A**). The results of the apoptosis analysis showed that AZD4547 alone did not promote apoptosis and nab-PTX alone increased cell apoptosis. However, AZD4547 combined with nab-PTX induced the most significant increase in cell apoptosis (**Figure 3B**). Furthermore, we observed changes in the expression of the apoptotic proteins Bax, Bcl-2, and cleaved caspase-3 in the different groups by Western blotting. The results showed that AZD4547 combined with nab-PTX led to increased apoptosis and activation of cleaved caspase 3. Moreover, Bax expression was increased and Bcl-2 expression was decreased in the combined treatment group (**Figure 3C**).

### Effect of the combination of AZD4547 and nab-PTX on gene expression, pathway activation, and biological processes

**Figure 4:**
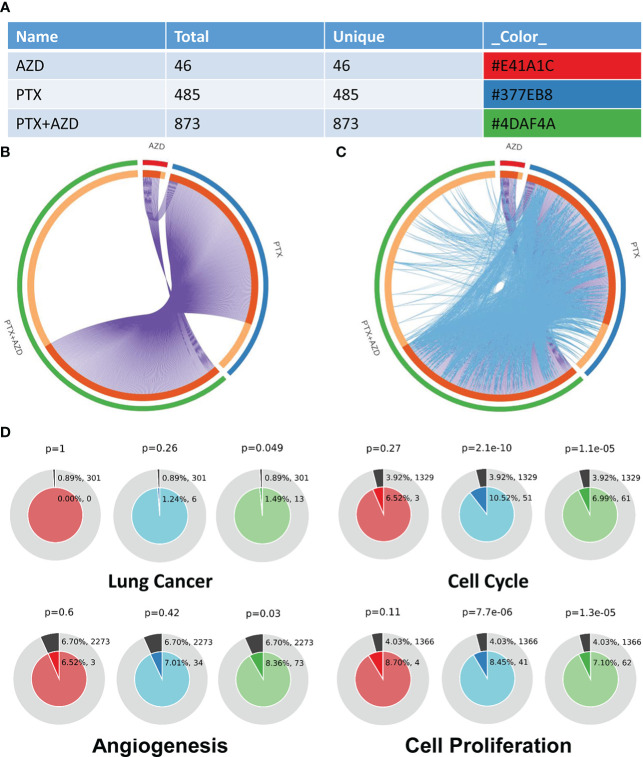
Gene annotation and membership search. **(A)** Gene List Summary. **(B, C)** Gene overlap analysis expanded *via* shared enriched ontologies. **(D)** Membership analysis: pie chart for visualization of membership enrichment (the outer pie shows the number and percentage of genes in the background associated with the membership (in black); the inner pie shows the number and percentage of genes in the individual input gene list associated with the membership).

We further investigated how treatment with AZD4547, nab-PTX, or AZD547 plus nab-PTX affected downstream signaling pathways by RNA-seq to explore the potential mechanism (**Supplementary Table 1**). All differentially expressed genes (DEGs) identifiers were first converted into their corresponding H. sapiens Entrez gene IDs using the latest version of the database (**Supplementary Tables 2, 3**). Each gene list was assigned a unique color that was used throughout the analysis, as summarized in **Figure 4A**. The overlaps between these lists are shown in a Circos plot, which shows data at only the gene level, and purple curves link identical genes (**Figure 4B**). Another valuable representation is to overlap genes based on their functions or shared pathways; in such a representation, blue curves link genes associated with the same enriched ontology term (**Figure 4C**). On the outside, each arc represents the identity of each gene list, according to the same color code used for the table rows in **Figure 4A**. The inner circle represents gene lists, and hits are arranged along the arc. Genes that overlap across multiple lists are colored in dark orange, and genes that are unique to a list are shown in light orange. In **Figures 4A–C**, we can see that the combination of AZD547 and nab-PTX affected more genes, functions or pathways.

A search for the membership term “Lung Cancer, Cell Cycle, Angiogenesis, Cell Proliferation” was applied to the following list of ontologies: Gene Ontology (GO) biological processes, GO cellular components, GO molecular functions, and Kyoto Encyclopedia of Genes and Genomes (KEGG) pathways. The p value indicates that genes related to lung cancer, cell cycle, angiogenesis, and cell proliferation were significantly enriched in the gene list (**Figure 4D**).

**Figure 5:**
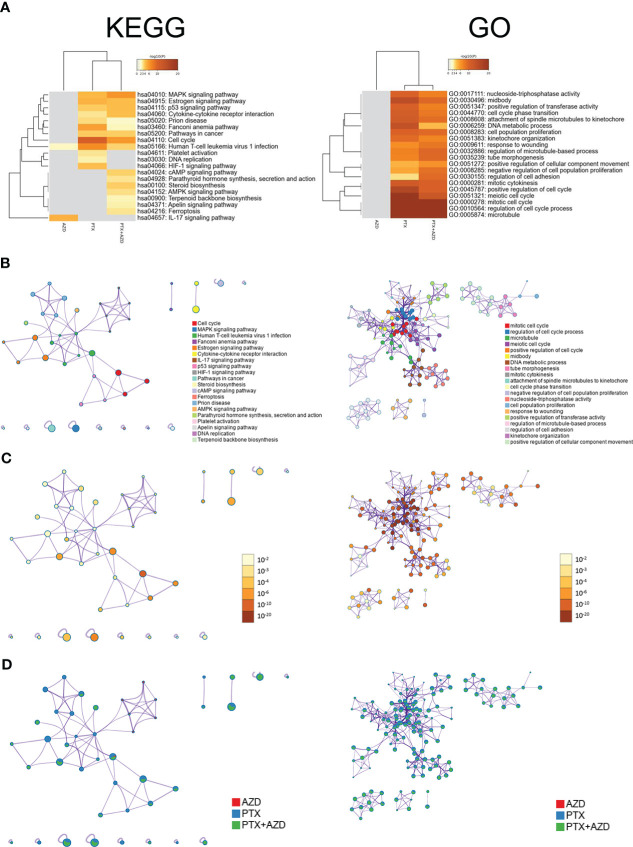
Functional enrichment analysis of DEGs (Left: KEGG. Right: GO). **(A)** Heatmap of enriched terms across input gene lists; the colors correspond to p values. The network of enriched terms is presented as follows: **(B)** colored based on cluster ID, where nodes that share the same cluster ID are typically close to each other; **(C)** colored by p value, where terms containing more genes tend to have a more significant p value; and **(D)** represented as pie charts, where pies are color-coded based on the identities of the gene lists.

For pathway and process enrichment analysis, we first identified all the significantly enriched KEGG and GO terms, and accumulative hypergeometric p values and enrichment factors were calculated and used for filtering. The remaining significant terms were then hierarchically clustered into a tree based on Kappa-statistical similarities among their gene memberships. We selected the term with the best p value within each cluster as its representative term and displayed these terms in a dendrogram (**Figure 5A**). To further analyze the relationships between terms, we selected a subset of representative terms from the full cluster and converted them into a network layout using Cytoscape. One term from each cluster was selected to have its term description shown as a label (**Figure 5B**). Additionally, the same enrichment network includes nodes whose colors are based on the p value, as shown in the legend (**Figure 5C**). The darker the color is, the more statistically significant the node is. Finally, the nodes of the enrichment network are displayed as pies (**Figure 5D**). Each pie sector is proportional to the number of hits originating from a gene list. KEGG pathway analysis revealed that compared with the single-drug group, the combined treatment group exhibited evident enrichment of the MAPK signaling pathway (**Supplementary Table 2**). GO analysis also revealed many altered and cancer-related enriched clusters, mainly related to the cell cycle, cell proliferation, cell adhesion, and angiogenesis (**Supplementary Table 3**).

### AZD4547 combined with nab-PTX inhibited phosphorylation of the MAPK signaling pathway

**Figure 6:**
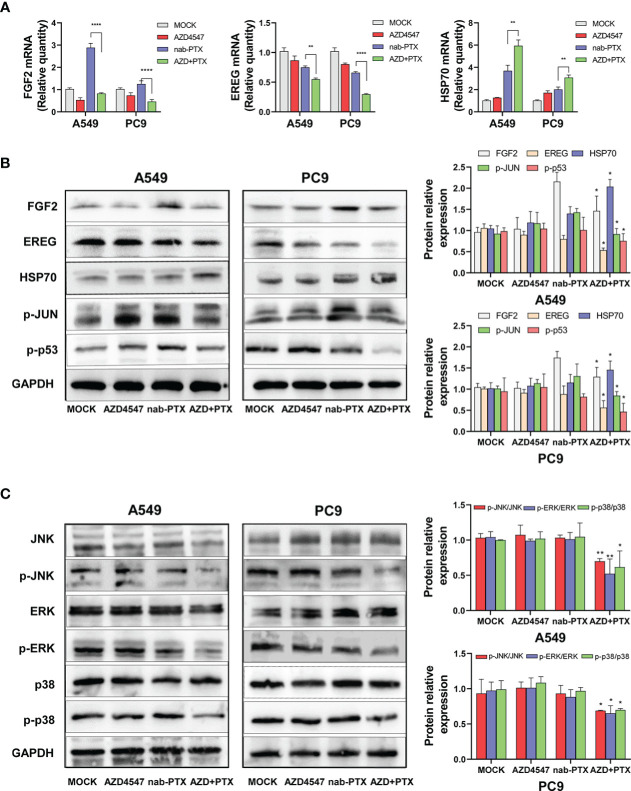
Effects of AZD4547 and nab-PTX on the MAPK signaling pathway. **(A)** The expression of FGF2, EREG, and HSP70 was assessed by qRT−PCR. **(B)** Western blot analysis of the protein expression of FGF2, EREG, and HSP70 and the phosphorylation of JUN and p53. **(C)** AZD4547 combined with nab-PTX inhibited phosphorylation of the JNK, ERK, and p38 proteins. *: p< 0.05, **: p< 0.01, ****: p< 0.0001.

Functional enrichment analysis of the DEGs that were identified by RNA-seq revealed that the DEGs in the combined treatment group showed significantly stronger enrichment of the MAPK signaling pathway than did those in the single-drug treatment group. For example, upstream of the MAPK pathway, five downregulated DEGs participated in activating the MAPK signaling pathway by functioning as growth factors (GFs: EREG, AREG, FGF2, BDNF, and TGFA), while four heat shock 70 kDa proteins (HSP70s: HSPA8, HSPA2, HSPA1A, and HSPA1B) among the upregulated DEGs were involved in inhibiting this pathway. Downstream of the pathway, the expression or modification of some transcription factors (TFs: JUN, FOS, and p53) also changed. To further confirm the sequencing results, the expression levels of some DEGs were evaluated by qRT−PCR (**Figure 6A**) and Western blot (**Figure 6B**). Compared with that of the nab-PTX alone group, the expression of EREG and FGF2 was significantly decreased in the combined treatment group, while the expression of HSP70 was further increased. Similarly, the phosphorylation of TFs (JUN and p53) downstream of the MAPK signaling pathway was also decreased.

Next, the JNK, ERK, and p38 MAPK pathways were analyzed by Western blot analysis (**Figure 6C**). Compared with nab-PTX alone, AZD4547 combination treatment caused no significant change in the expression of JNK, ERK, or p38. However, the phosphorylation of JNK, ERK, and p38 was significantly decreased in the combined treatment group. These results revealed that AZD4547 combined with nab-PTX led to inhibition of the JNK, ERK, and p38 MAPK pathways. The MAPK signaling pathway may mediate the synergistic antitumor effect of AZD4547 combined with nab-PTX.

## Discussion

The deregulation of FGFR signaling plays a vital role in cancer development and progression. In approximately 20% of NSCLC patients, FGFR1 is overexpressed, indicating the potential of FGFR to serve as a therapeutic target in cancer treatment (15–17). However, preclinical studies have shown that AZD4547 exerts no effects against many tumor cell lines without deregulated FGFR expression, such as the A549 and PC9 cell lines. Recent clinical trials have also confirmed that most patients may not respond well to FGFR inhibitors (4–9). How to maximize the antitumor effects of FGFR inhibitors warrants further exploration. Our data are consistent with a previous report; the IC50 values for AZD4547 in A549 and PC9 cells were 24.46 μM and 24.09 μM, respectively (3). However, in a series of further experiments, we were surprised to find that AZD4547 enhanced the sensitivity of NSCLC cell lines without deregulated FGFR expression to nab-PTX. Consistently, *in vivo* and *in vitro* experiments showed that the combination of AZD4547 with nab-PTX exerted synergistic antitumor effects. Our results illustrated that AZD4547 alters the regulatory effect of nab-PTX on cell cycle progression and apoptosis. Western blotting assays showed that the apoptosis pathway was activated. The combination of AZD4547 and nab-PTX promoted an imbalance in Bax and Bcl-2 expression, increased the cleavage of caspase-3, and ultimately led to apoptosis.

To further explore the possible mechanism by which AZD4547 enhances nab-PTX-mediated growth inhibition in lung cancer cells, we conducted RNA-seq and bioinformatics analysis. Our results showed that low-dose AZD4547 had little effect on the transcriptome of A549 cells, but the addition of AZD4547 significantly altered the effect of nab-PTX on the transcriptome, and the most noticeable change was in the MAPK signaling pathway.

MAPK cascades, which mainly include the extracellular responsive kinase (ERK), c-Jun N-terminal kinase (JNK), and p38 MAPK pathways, are complex. The MAPK signaling pathway is essential for initiating a wide array of cellular processes in response to extracellular stimuli. A number of growth factor receptors, including fibroblast growth factor receptors (FGFRs), are upstream of the MAPK pathways (18). Therefore, the MAPK pathways are regulated by various growth factors (GFs), such as peptide growth factors, cytokines, and hormones, and in turn regulate cell proliferation, differentiation, and survival (19). The Raf-MEK-ERK signaling pathways are known to transmit signals from GF receptors and further modulate gene expression, possibly preventing apoptosis (20). Recent research showed that the JNK signaling pathway performs dual functions by regulating cell apoptosis and survival. The potential molecular mechanisms by which JNK promotes cancer cell survival include inducing autophagy, inhibiting cell apoptosis, promoting tumor immune evasion, activating compensatory cell proliferation, and participating in crosstalk with other signaling factors (including NF-κB, p38, and JunD) (21). Numerous studies have shown that p38 performs pro-oncogenic functions in various types of cancer. P38 MAPK pathway activation is triggered by a wide range of stimuli, including environmental stimuli, genotoxic and DNA-damaging agents, growth factors, and cytokines. Upon activation, p38 MAPK regulates multiple cellular functions ranging from cell proliferation to cell apoptosis, cell invasion, and survival. Therefore, the p38 MAPK signaling pathway has been proposed as a critical node in cancer and cancer treatment (22–24).

Our results showed that AZD4547 combined with nab-PTX could regulate the genes that are involved in the MAPK pathway and inhibit the phosphorylation of JNK, ERK, and p38. The inhibition of the MAPK signaling pathway may be the potential mechanism by which AZD4547 enhances the sensitivity of NSCLC cells without deregulated FGFR expression to nab-PTX, regulates cell cycle progression, promotes apoptosis, and inhibits cell proliferation.

Drug resistance has always been a serious clinical problem that causes high mortality rates in patients with advanced cancer. Similar to other targeted drugs, FGFR inhibitors are limited by the development of drug resistance. One of the causes of resistance to FGFR inhibitors is the activation of alternative pathways, such as the MAPK signaling pathway (25). For example, Izabela Zarczynska et al. found that the p38 MAPK pathway is responsible for FGFR inhibitor resistance, and drug-resistant variants of FGFR inhibitor-sensitive cells are characterized by increased p38 expression/phosphorylation as well as enhanced expression of genes that are involved in MAPK signaling (26). The identification of resistance mechanisms and alternative pathways provides a basis for the use of combination therapy strategies. Combination therapy is increasingly likely to be required for cancer treatment, as such approaches improve efficacy and may prevent or delay the occurrence of drug resistance. Therefore, blocking both FGF/FGFR and MAPK signaling provides a way to bypass resistance to FGFR inhibitors (27, 28).

Paclitaxel is widely recognized as an antitumor drug, as it prevents mitotic progression by stabilizing microtubules. Recent reports have shown that paclitaxel can also inhibit the proliferation of cancer cells through the EGFR/MAPK signaling pathway (29, 30). Therefore, the combination of AZD4547 and nab-PTX may target multiple pathways simultaneously, which can effectively increase antitumor efficacy and synergistically prevent the occurrence of drug resistance. However, it should be noted that MAPK cascades are complex signaling pathways; therefore, different types of tumors or cells, drug combinations, and even administration times and doses may yield different results. In addition, many critical issues still need to be resolved, including how to identify the patients who are most likely to benefit from treatment and how to determine the best combination of FGFR inhibitors with other chemotherapies or biological agents to maximize their antitumor effect and avoid or at least delay resistance development as much as possible. Further studies and trials are still needed.

## Conclusion

**Figure 7:**
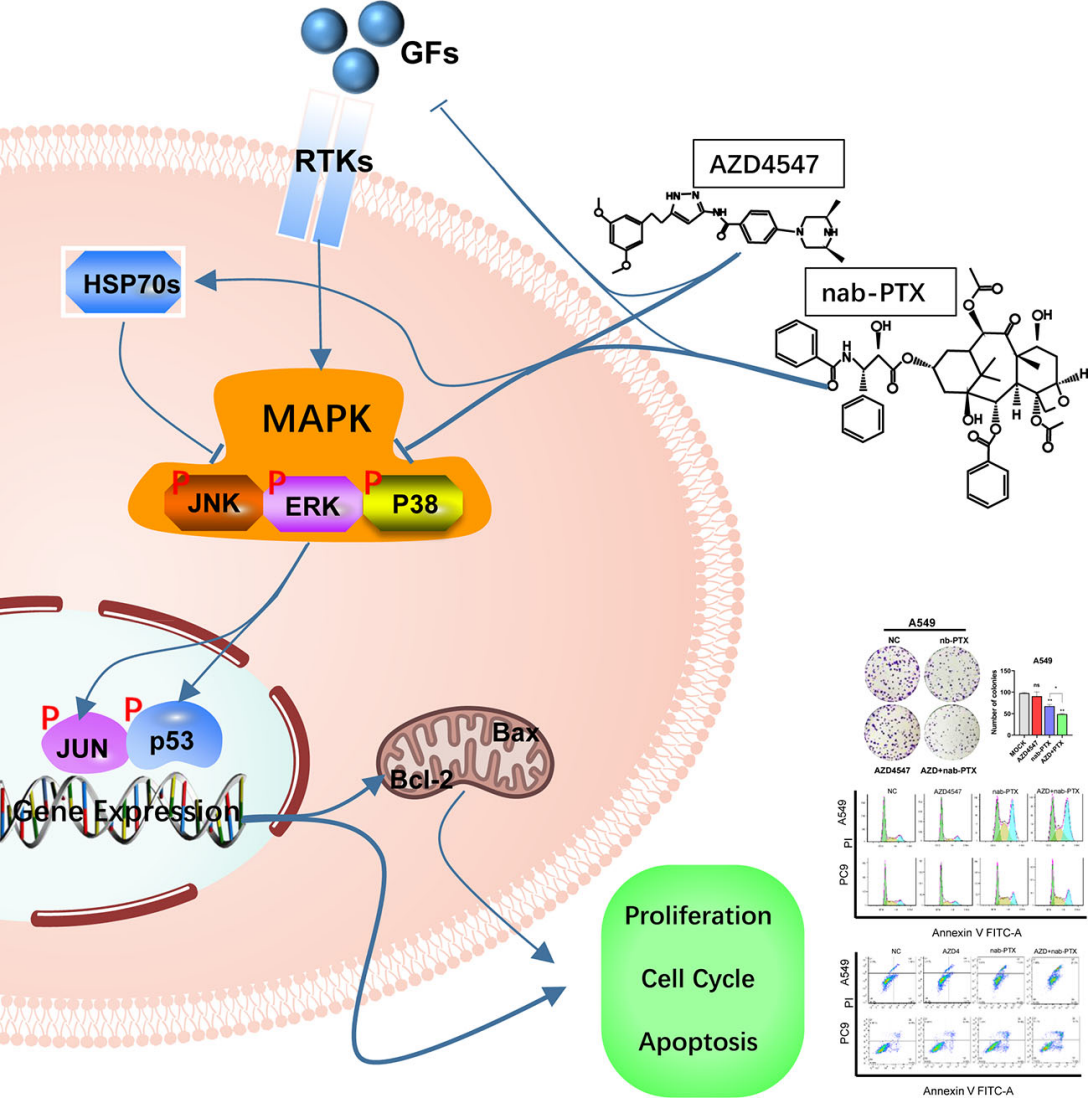
The combination of AZD4547 and nab-PTX inhibited cell proliferation, led to cell cycle arrest in the G2/M phase, promoted apoptosis, and exerted a synergistic antitumor effect by suppressing phosphorylation of MAPK signaling pathway components (GFs, growth factors; RTKs, receptor tyrosine kinases; HSP70s, heat shock 70 kDa proteins).

AZD4547 combined with nab-PTX suppressed the phosphorylation of MAPK signaling pathway components, which led to cell cycle arrest in the G2/M phase, promoted apoptosis, and exerted a profound inhibitory effect on cell proliferation (**Figure 7**). Our findings might provide a new strategy for the rational use of FGFR inhibitors and personalized treatment of NSCLC patients.

## Data availability statement

The datasets presented in this study can be found in online repositories. The names of the repository/repositories and accession number(s) can be found in the article/**Supplementary Material**.

## Ethics statement

The animal study was reviewed and approved by Animal Care and Use Committee of Zhujiang Hospital, Southern Medical University.

## Author contributions

MX, FM, and XZ conceived and designed the experiments. FM, YX, and YD performed the experiments. FM, YN, and AN analyzed the data. FM and YN wrote the paper. SB revised the manuscript. MX, JS, and YL acquired funding and performed administration and supervision. The first three authors contributed equally to this paper. All authors contributed to the article and approved the submitted version.
